# Melatonin ameliorates heat stress-induced oxidative apoptosis in mouse spermatocytes *via* autophagy and ferroptosis pathways

**DOI:** 10.1016/j.cstres.2025.100078

**Published:** 2025-04-20

**Authors:** Yi-Ping Lei, Jia Wang, Peng-Luo Yin, Hua Jia, Wen-Zhi Ma

**Affiliations:** Key Laboratory of Fertility Preservation and Maintenance of the Ministry of Education, Key Laboratory of Reproduction and Genetics of Ningxia Hui Autonomous Region, School of Basic Medical Science, Ningxia Medical University, Yinchuan 750004, China

**Keywords:** Melatonin, Spermatocyte injury, Testicular heat stress, Ferroptosis, Autophagy regulation

## Abstract

Testicular heat stress is a critical factor contributing to male infertility, with spermatocytes exhibiting heightened sensitivity to temperature elevation. This study systematically elucidates the protective mechanisms of melatonin against heat stress-induced spermatocyte injury. In a murine heat stress model, melatonin intervention significantly reduced testicular accumulation of malondialdehyde (MDA) induced by heat stress, enhanced the activities of catalase (CAT) and superoxide dismutase (SOD), and suppressed germ cell apoptosis by downregulating the pro-apoptotic protein Bax and upregulating GPX4 expression. Sycp3 immunohistochemistry demonstrated that melatonin significantly improved spermatocyte structural integrity. In the GC-2spd (ts) spermatocyte cell line model, melatonin treatment markedly reduced MDA levels and alleviated heat stress-induced oxidative apoptosis and proliferation inhibition by downregulating key apoptotic proteins (Bax, Caspase-3, and cleaved-Caspase-3). Mechanistic studies revealed that melatonin restores autophagic balance by modulating the expression of autophagy-related proteins LC3-I, LC3-II, and P62. Concurrently, melatonin downregulated ferroptosis markers P53 and COX2, inhibiting ferroptosis by blocking DNA damage response and inflammatory amplification pathways. Melatonin synergistically maintained cellular redox homeostasis by downregulating the NRF2/HO-1 pathway and upregulating GPX4 expression, significantly reducing Fe²⁺ accumulation and ameliorating iron metabolism dysregulation. This study unveils the molecular mechanisms by which melatonin mitigates testicular heat stress injury through a multitarget regulatory network, providing novel therapeutic strategies for clinical intervention in heat stress-associated infertility.

## Introduction

Infertility affects 10–20% of the global reproductive-age population, with approximately 30–50% of cases directly attributed to male reproductive dysfunction.^1^ This health issue not only imposes psychological burdens on individuals but also exerts significant socioeconomic impacts. Among various pathogenic factors, testicular hyperthermia has emerged as a critical risk factor for male infertility due to its broad environmental and pathological relevance.^2^ Clinical studies confirm that prolonged sitting, occupational heat exposure,^3^ varicocele, and cryptorchidism can elevate testicular temperature, disrupting the homeostasis of the spermatogenic microenvironment.^4^ The physiological temperature of mammalian testes is typically 2–8 °C lower than core body temperature, a unique thermoregulatory mechanism essential for normal spermatogenesis.^5^, ^6^ Notably, pachytene/diplotene spermatocytes and early round spermatids during meiosis are particularly sensitive to temperature fluctuations.^7^ Heat stress triggers a cascade of reactions through oxidative stress (OS), ultimately leading to germ cell loss and structural damage to the seminiferous epithelium, though the molecular mechanisms remain incompletely elucidated.

Testicular injury induced by heat stress is closely associated with OS.^8^ When local testicular temperature rises, dysfunction of the mitochondrial electron transport chain leads to excessive generation of reactive oxygen species (ROS), disrupting intracellular oxidative/antioxidant balance.^9^, ^10^ Studies have demonstrated that heat stress significantly increases ROS production in testicular spermatogonia, resulting in oxidative damage. ROS promotes structural destruction of cells and triggers oxidative apoptosis of germ cells by activating both p53-dependent and -independent Fas/FasL pathways and other apoptotic signaling cascades.^11^ Excessive ROS disrupts autophagic homeostasis. Specifically, ROS activates the Adenosine Monophosphate-Activated Protein Kinase pathway while inhibiting mTOR (mTOR) signaling, thereby inducing autophagosome formation. Oxidative modification of autophagy-related proteins (e.g, Atg) blocks LC3-II lipidation, leading to impaired autophagic flux,^12^ accumulation of damaged organelles, and further exacerbation of OS. Heat stress-induced lipid peroxidation synergizes with iron overload to trigger ferroptosis. In porcine Sertoli cells, heat stress-evoked OS generates substantial lipid peroxides and promotes ferroptosis *via* the CYP2C9-Ras-JNK signaling axis.^11^, ^12^ Excessive ROS production causes cellular damage through multiple pathways, including apoptosis, autophagy, DNA damage, and ferroptosis.^13^ Therefore, in-depth exploration of the mechanisms underlying heat stress-induced lipid peroxide accumulation and subsequent cell death is crucial for understanding its impact on male germ cells.

Melatonin (N-acetyl-5-methoxytryptamine) is an endogenous antioxidant molecule synthesized in the pineal gland and testes.^14^, ^15^ It has been shown to effectively prevent various ROS-related diseases by activating antioxidant enzyme systems, scavenging ROS, and inhibiting inflammatory responses.^16^, ^17^, ^18^ In the reproductive system, melatonin exhibits multidimensional protective effects. Studies have demonstrated that, in a chemotherapeutic agent-induced mouse model of testicular injury, melatonin alleviates OS-induced apoptosis in spermatogonial stem cells by scavenging ROS and inhibiting the p38 signaling pathway, promoting the expression of MnSOD and SIRT1.^16^ Moreover, in a hydroxy camptothecin-induced testicular injury model, melatonin protects testicular cells by activating autophagy to alleviate OS and mitochondrial dysfunction.^17^ In an acute kidney injury model, melatonin significantly reduces lipid peroxide levels by inhibiting the NRF2/Slc7a11 axis-mediated ferroptosis.^18^ Despite melatonin's significant reproductive protective effects in various pathological conditions, its potential to regulate autophagy, iron metabolism, and ROS interactions in heat stress models and its protective effects on spermatocytes remain to be explored.

This study aims to investigate the roles of autophagy and ferroptosis in heat stress-induced testicular injury and further explore whether melatonin alleviates heat stress-triggered oxidative apoptosis in spermatocytes by inhibiting these pathways, thereby preventing testicular damage. Our findings aim to provide novel therapeutic strategies for heat stress-associated reproductive disorders and establish a theoretical foundation for the application of melatonin in reproductive health.

## Results

### Heat stress induces apoptosis in testicular germ cells

**Fig. 1:**
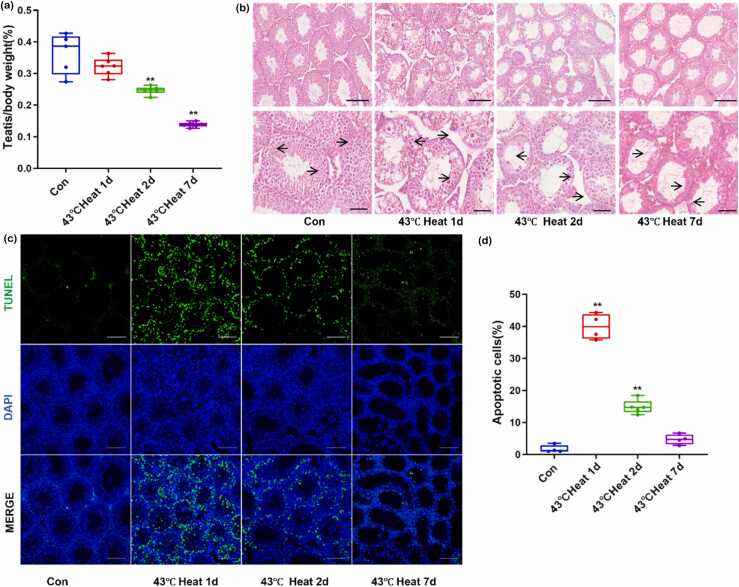
Heat stress induces germ cell apoptosis and causes testicular damage. (a) Testis-to-body weight ratio at different time points following heat stress. (b) Hematoxylin and eosin staining of testicular seminiferous tubules showing structural changes postheat stress. Scale bar = 100 µm (upper panel) and 50 µm (lower panel). (c) and (d) Quantification of TUNEL-positive cells in each group. Scale bar = 100 µm. In (a) and (d), values are compared to the control group (Con), *P* < 0.01. TUNEL, Terminal deoxynucleotidyl transferase dUTP Nick-End Labeling; DAPI, 4',6-Diamidino-2-Phenylindole.

The testicular organ index demonstrated that testicular weight and size decreased progressively with increasing doses of heat stress. At 1, 2, and 7 days postheat stress, the testicular organ index in mice was significantly lower than in the control (Con) group, reaching its minimum value at 7 days post treatment (Figure 1(a)). Hematoxylin and eosin (H&E) staining revealed intact overall morphology of seminiferous tubules in the Con group, with orderly arrangement of spermatogenic cells at all stages. At 1 day postheat stress, cells exhibited enlarged cytoplasm and nuclei resembling round spermatids, alongside clusters of spermatid-like nuclei. By day 2, most seminiferous epithelia showed degenerative changes and vacuolization. At day 7, extensive vacuolization and loss of germ cell layers were observed in the majority of tubules (Figure 1(b)). Terminal deoxynucleotidyl transferase dUTP Nick-End Labeling (TUNEL) assay detected abundant TUNEL-positive cells in testes 1 day after 43 °C heat stress, with a gradual reduction in their numbers over time. Spatial analysis indicated that TUNEL-positive cells were initially localized near the basal compartment of the tubules and progressively expanded toward the lumen (Figure 1(c)). Collectively, these findings confirm that heat stress triggers germ cell apoptosis, leading to testicular injury.

### Melatonin effectively ameliorates testicular damage induced by OS

**Fig. 2:**
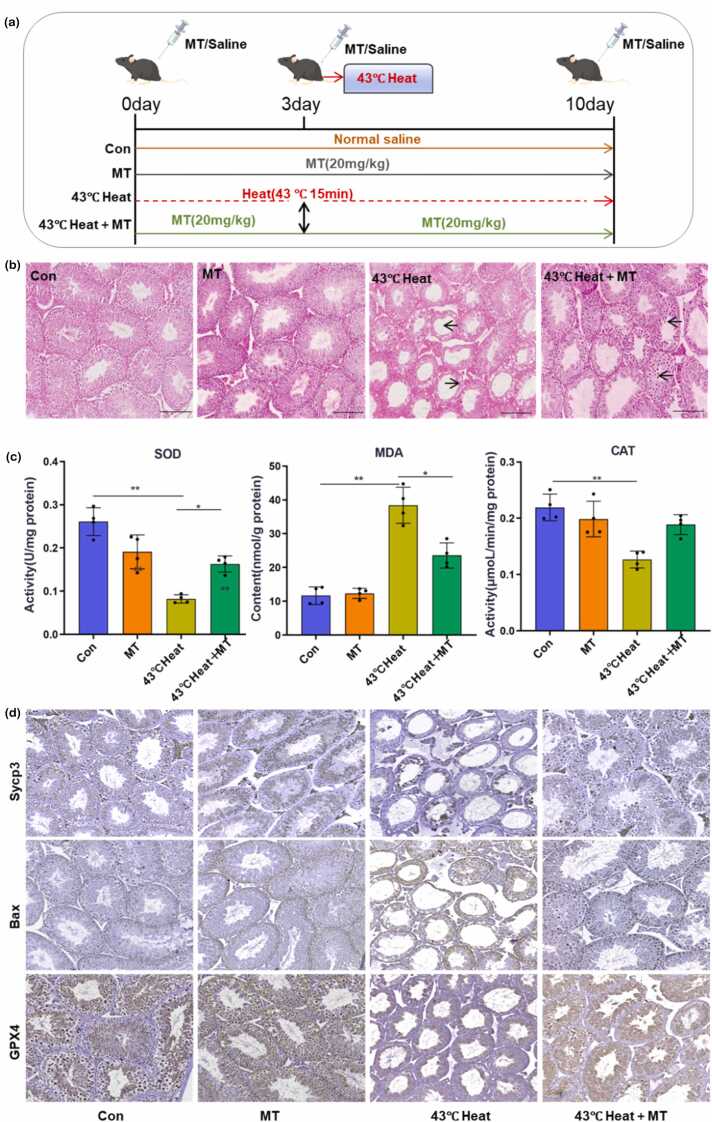
Melatonin ameliorates testicular damage induced by heat stress. (a) Experimental protocol for melatonin and heat stress interventions in 6-week-old male mice. (b) Hematoxylin and eosin staining analysis of structural changes in the seminiferous tubules of testicular tissues from different groups. Scale bar = 100 µm. (c) Measurement of SOD, MDA, and CAT levels in testicular homogenates from different groups. Pairwise comparisons between groups: *P < 0.05, **P < 0.01. (d) Immunohistochemical analysis of Sycp3, Bax, and GPX4 expression and localization in testicular tissue sections. Data are representative of three independent experiments. Scale bar = 100 µm. Abbreviations used: GPX4, glutathione peroxidase 4; MDA, malondialdehyde; CAT, catalase; SOD, superoxide dismutase.

Previous studies have reported that melatonin protects testicular cells against adverse conditions.^19^, ^20^ To determine whether melatonin ameliorates heat stress-induced testicular damage, we administered melatonin at a dose of 20 mg/kg/d. The MT and 43 °C heat+MT groups received intraperitoneal melatonin injections starting 3 days prior to heat stress and continuing until 7 days post treatment. The control (Con) and 43 °C heat groups were injected with an equivalent volume of saline (Figure 2(a)). H&E staining of testicular tissues revealed that melatonin (MT group) did not affect the morphology or cellular arrangement of seminiferous tubules. In contrast, the 43 °C heat group exhibited significant germ cell loss within the tubules, while the 43 °C heat+MT group showed marked improvement in vacuolization caused by germ cell depletion (Figure 2(b)). Consistent with prior findings that heat stress exacerbates testicular OS, the 43 °C heat group displayed significantly elevated malondialdehyde (MDA) levels and reduced catalase (CAT) and superoxide dismutase (SOD) activities compared to the Con group (*P* < 0.01). In the 43 °C heat+MT group, MDA levels were significantly lower, while SOD and CAT activities were notably higher than in the 43 °C heat group (*P* < 0.05) (Figure 2(c)). Sycp3 immunohistochemistry demonstrated that melatonin significantly improved spermatocyte structural integrity. Immunohistochemical analysis of the apoptotic protein Bax revealed that 43 °C heat stress-induced substantial upregulation of Bax in spermatocytes, which was attenuated in the 43 °C heat+MT group. Glutathione peroxidase 4 (GPX4), a key enzyme in the antioxidant system, showed significantly reduced expression in the 43 °C heat group compared to the control group. However, GPX4 expression was restored in the 43 °C heat+MT group (Figure 2(d)).

### Melatonin alleviates heat stress-induced germ cell proliferation inhibition and apoptosis

**Fig. 3:**
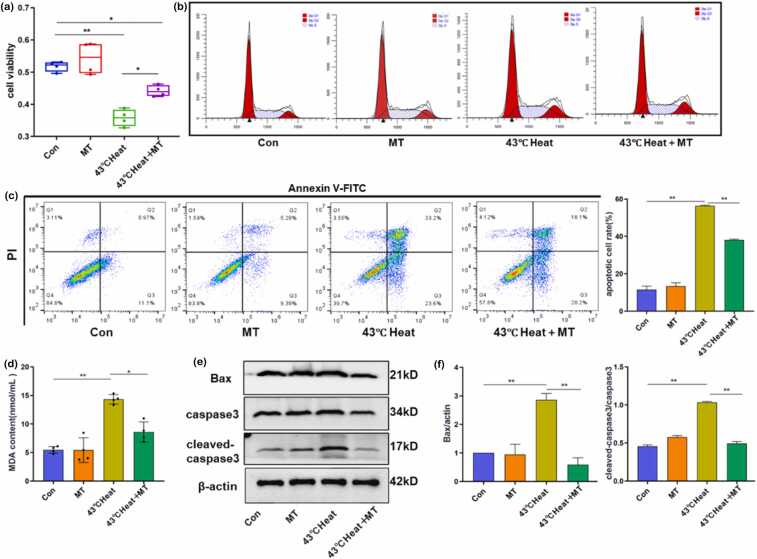
Melatonin alleviates heat stress-induced germ cell proliferation inhibition and apoptosis. (a) CCK8 assay showing cell viability across different treatment groups. (b) Flow cytometric analysis of cell cycle distribution (G1-S-G2/M phases) in different groups. (c) Flow cytometric analysis of apoptosis rates in GC-2 cells under various treatments. (d) Measurement of MDA levels in testicular homogenates from different groups. (e and f) Western blot analysis of apoptosis-related protein expression in testes from different treatment groups. In panels (c) and (d), pairwise comparisons between groups: *P < 0.05, **P < 0.01. Abbreviations used: MT, melatonin; MDA, malondialdehyde.

Cell viability assays revealed no significant difference in spermatocyte viability between the MT and Con groups. However, spermatocyte viability in the 43 °C heat group was significantly reduced compared to the control group (*P* < 0.01). In the 43 °C heat+MT group, viability was notably higher than in the 43 °C heat group (*P* < 0.05), though it did not fully recover to Con group levels (Figure 3(a)).

Flow cytometry results indicated no significant differences in the proportions of cells in G0/G1, S, or G2/M phases between the MT and Con groups. In contrast, the 43 °C heat group showed a reduced proportion of cells in the G0/G1 phase and an increased proportion in the S phase compared to the Con group (*P* < 0.05), suggesting that heat stress induces S-phase arrest and disrupts cell cycle progression. The 43 °C heat+MT group exhibited restored normal cell cycle distribution (Figure 3(b)).

Cell cycle arrest caused by heat stress further promoted apoptosis, a critical biological process for maintaining tissue homeostasis. Annexin V-FITC/PI double staining flow cytometry demonstrated a significant increase in apoptosis rates in the 43 °C heat group compared to the Con group (*P* < 0.05), while the 43 °C heat+MT group showed reduced apoptosis rates relative to the 43 °C heat group (*P* < 0.05) (Figure 3(c)). To confirm these findings, we analyzed the levels of apoptosis-related proteins in GC-2 cells. As shown in Figure 3(d), melatonin (MT) significantly attenuated the heat stress-induced upregulation of Caspase-3, cleaved-Caspase-3, and Bax in GC-2 cells (Figure 3(d)).

### Transcriptomic analysis reveals the mechanism by which melatonin alleviates heat stress-induced spermatogonial apoptosis

**Fig. 4:**
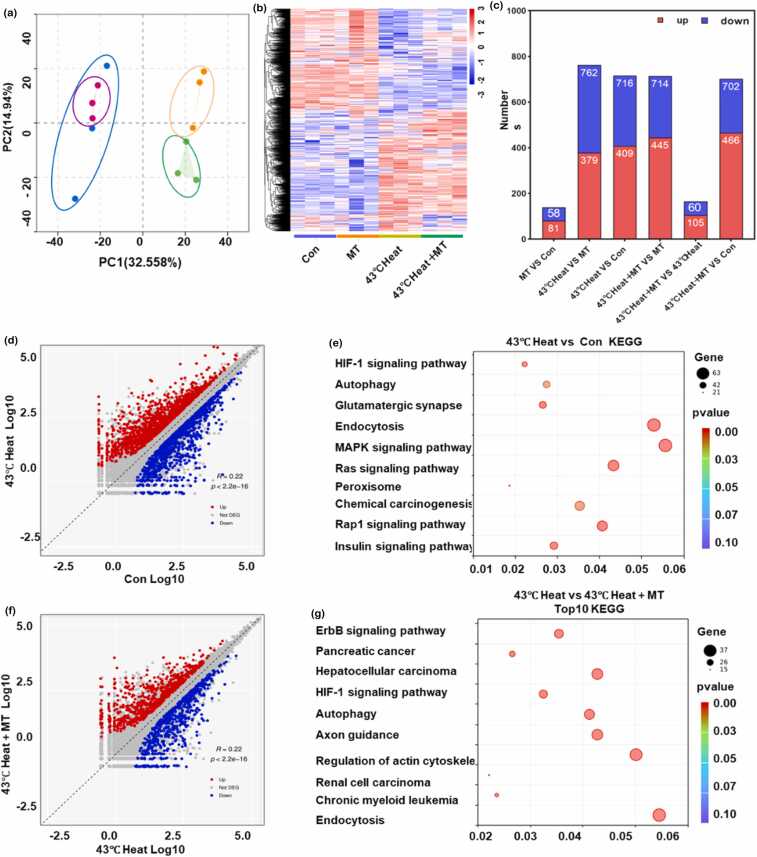
Transcriptomic analysis reveals the regulatory mechanism of melatonin and heat stress in germ cells. (a) Principal component analysis illustrating the intergroup differences between the sample groups. (b) and (c) Heatmap and bar chart showing the number of differentially expressed genes in each group. (d) and (e) Scatter plot and bubble plot analyzing the significantly differentially expressed genes between the Con and 43 °C heat groups and their enriched KEGG pathways. (f) and (g) Scatter plot and bubble plot analyzing the significantly differentially expressed genes between the Con and 43 °C heat groups, along with the top 10 enriched KEGG pathways. Abbreviation used: MT, melatonin; KEGG, Kyoto Encyclopedia of Genes and Genomes.

To elucidate the mechanisms by which melatonin alleviates heat stress-induced spermatogonial apoptosis, principal component analysis was performed to assess intergroup significance among the Con, MT, 43 °C heat, and 43 °C heat+MT groups. The results demonstrated a clear separation between the 43 °C heat and 43 °C heat+MT groups compared to the control (Figure 4(a)). RNA sequencing (RNA-seq) was conducted to analyze the gene expression profiles across these groups. Differentially expressed genes (DEGs) were identified using thresholds of Q ≤ 0.05 and |fold change| > 1. Compared to the Con group, 716 DEGs (409 upregulated and 307 downregulated) were detected in the 43 °C heat group. In contrast, 165 DEGs (105 upregulated and 60 downregulated) were identified between the 43 °C heat+MT and 43 °C heat groups (Figure 4(b)).

Kyoto Encyclopedia of Genes and Genomes pathway enrichment analysis further annotated DEGs associated with heat stress-induced germ cell apoptosis, revealing significant enrichment in pathways related to OS, autophagy, cell signaling, and metabolic dysregulation. Gene ontology analysis of the top 10 enriched pathways between the 43 °C heat and 43 °C heat+MT groups highlighted autophagy, Mitogen-Activated Protein Kinase signaling, and Erythroblastic Leukemia Viral Oncogene Homolog signaling pathways (Figure 4(c)). These findings suggest that melatonin mitigates heat stress-induced spermatogonial apoptosis by enhancing antioxidant defenses, suppressing lipid peroxidation, and regulating autophagy to reduce ferroptosis (Figure 4(d,e,f,g)).

### Melatonin improves heat stress-induced spermatocyte apoptosis by inhibiting autophagy and ferroptosis pathways

**Fig. 5:**
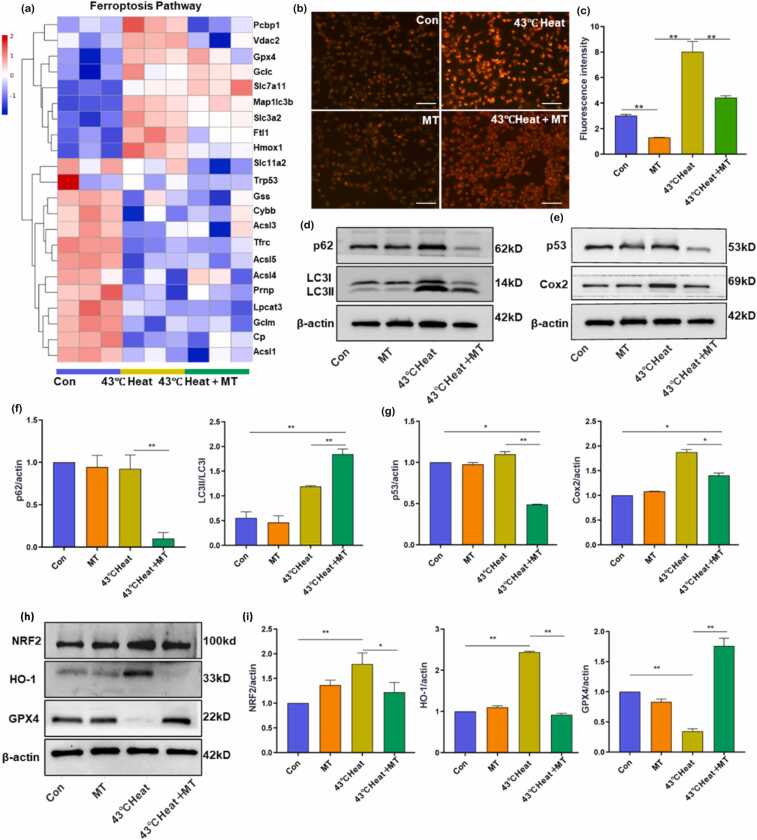
Melatonin alleviates heat stress-induced apoptosis in GC-2 cells via autophagy and ferroptosis pathways. (a) Heatmap showing differentially expressed genes involved in the ferroptosis pathway between Con, 43 °C heat, and 4C heat + MT groups. (b) and (c) Changes in intracellular Fe²⁺ levels in cells from each group. (d) and (f) Western blot analysis of autophagy-related proteins p62 and LC3-II/LC3-I expression. (e, g, h, i) Western blot analysis of key proteins involved in ferroptosis: p53, Cox2, NRF2, HO-1, and GPX4. Data are presented as mean ± SD. Con versus 43 °C heat: *P < 0.05, **P < 0.01. 43 °C. Heat versus 43 °C heat + MT: *P < 0.05, **P < 0.01. Abbreviations used: MT, melatonin; GPX4, glutathione peroxidase 4.

To better capture the changes in ferroptosis-related gene expression, a heatmap was constructed for visualization. Compared to the control group, heat stress significantly upregulated the expression of ferroptosis-related genes, including Pcbp1, Gpx4, FTL1, Ggt6, and Hmox1, while the expression of Acsl3 was downregulated. RNA-seq analysis indicated that ferroptosis is involved in heat stress-induced testicular damage (Figure 5(a)). Ferro Orange staining results showed a significant increase in fluorescence intensity in the 43 °C heat group compared to the control group, suggesting that heat stress markedly elevates ferroptosis levels in spermatocytes. In the 43 °C heat + MT group, fluorescence intensity was notably reduced compared to the 43 °C heat group, indicating that melatonin effectively alleviates heat stress-induced ferroptosis in spermatocytes. Elevated levels of Fe²⁺ can generate highly reactive hydroxyl radicals through the Fenton reaction, leading to the accumulation of lipid peroxides (Figure 5(b)).

To further validate these findings, we performed Western blot analysis of OS-related protein and autophagy-related protein in cells collected from the Con, MT, 43 °C heat, and 43 °C heat + MT groups. The results revealed that heat stress significantly upregulated the expression of autophagy-related proteins LC3-I, LC3-II, and P62. In the 43 °C heat + MT group, the expression of LC3-I, LC3-II, and P62 was reduced compared to the 43 °C heat group (Figure 5(d)). Ferroptosis-related protein analysis showed that heat stress induced a marked increase in the expression of p53, COX2, Nrf2, and HO-1, while GPX4 expression was significantly downregulated. In the 43 °C heat + MT group, the expression of p53, COX2, Nrf2, and HO-1 was reduced, while GPX4 expression was significantly upregulated (Figure 5(e) and (h)).

## Discussion

In recent decades, frequent global extreme heat events have exerted profound impacts on ecosystems, human health, and economies.^21^ High-temperature environments disrupt thermoregulatory mechanisms, leading to abnormal elevation of core body temperature due to impaired heat dissipation.^22^ Male germ cells are highly sensitive to heat stress, with pachytene and diplotene spermatocytes being particularly vulnerable to damage, thereby contributing to male infertility.^23^, ^24^ Melatonin, a potent antioxidant, has been demonstrated to alleviate OS in multiple organs,^25^ promote spermatogonial stem cell proliferation, and protect murine spermatocytes from drug-induced apoptosis. In this study, melatonin significantly attenuated heat stress-induced OS-induced apoptosis in spermatocytes across both in vivo and in vitro models. Further mechanistic investigations revealed that melatonin effectively mitigates testicular injury caused by heat stress through synergistic regulation of OS, autophagic homeostasis, and ferroptosis pathways.

It is noteworthy that conventional whole-body hyperthermia models may compromise the accuracy of mechanistic interpretation due to secondary inflammatory responses in visceral organs.^26^ In contrast, we innovatively developed a localized heat stress model by immersing the lower limbs and scrotum of anesthetized mice in a 43 °C water bath for 15 min. This approach specifically induced scrotal tissue damage while maintaining core body temperature homeostasis, thereby simulating the pathophysiological scenario where thermoregulatory mechanisms stabilize systemic temperature despite localized hyperthermal exposure in the scrotum under high ambient temperatures.^6^, ^27^ Testicular injury caused by heat stress is closely associated with OS.^28^, ^29^ In pathological conditions such as cryptorchidism and varicocele, ROS levels in testicular tissues are also significantly elevated. Previous studies have confirmed the protective effects of antioxidants like taurine, quercetin, and glutamine against testicular oxidative damage.^30^, ^31^ Under OS, melatonin maintains the viability of nucleus pulposus cells, reduces apoptosis rates, and decreases MDA levels.^32^ Our findings demonstrate that melatonin significantly increases the levels of antioxidant enzymes SOD and CAT while reducing MDA content (Figure 2(c)). Concurrently, melatonin enhances GPX4 expression (Figure 2(d)), suppresses lipid peroxidation, and restores the balance between ROS and antioxidants under heat stress, thereby effectively protecting the male reproductive system. Furthermore, we showed that melatonin treatment preserves testicular histology and improves the testicular index, promoting recovery from thermal injury, which is critical for restoring fertility postheat stress (Figures 1(a) and 2(b)). Therefore, utilizing melatonin to mitigate heat stress-induced testicular oxidative damage represents a vital strategy for safeguarding normal testicular cell development.

Studies have shown that spermatocytes are particularly vulnerable to heat stress-induced damage.^23^, ^33^ Hutson *et al*.^34^ reported that mouse spermatocytes exposed to 35 °C for 24 h exhibited a significant increase in DNA fragmentation.^34^ Spermatocytes are more susceptible to lipid peroxidation damage compared to round spermatids,^35^, ^36^ rendering them highly sensitive to OS caused by heat stress. In this study, we observed a substantial loss of spermatocytes following heat stress, effectively mitigated by melatonin intervention (Figure 2(d)). As a potent antioxidant, melatonin reduces spermatogonial apoptosis induced by BPDE by activating the Nrf-2/ARE antioxidant pathway and suppressing ROS levels in spermatogonia.^37^ Additionally, melatonin enhances testicular antioxidant capacity, promotes testosterone synthesis, and attenuates BPA-induced OS and testicular cell apoptosis.^38^ Our findings further validate that melatonin inhibits heat stress-triggered oxidative apoptosis in spermatocytes (Figure 3(b) and (c)), evidenced by reduced MDA content and downregulated expression of apoptosis-related proteins, including Caspase-3, cleaved-Caspase-3, and Bax (Figure 3(e)), consistent with prior research.

Melatonin, as a potent antioxidant, regulates cellular redox homeostasis through multiple mechanisms: directly scavenging ROS and enhancing cellular defense by activating endogenous antioxidant systems (e.g., SOD, CAT). Notably, its antioxidant functions are closely linked to autophagy modulation. Studies indicate that melatonin maintains cellular homeostasis by suppressing FOXO1-mediated excessive autophagy activation while protecting murine granulosa cells from oxidative damage. In human retinal pigment epithelial cell models, melatonin significantly alleviates H₂O₂-induced oxidative injury by upregulating autophagy markers (LC3-II and Beclin-1) and promoting p62 degradation.^39^ This study further reveals that heat stress abnormally activates autophagy *via* OS; however, the aberrant accumulation of autophagosome markers (LC3-II) and substrate proteins (p62) suggests impaired lysosomal function and subsequent autophagic flux blockade (Figure 5(d)). This observation aligns with prior findings: heat stress-induced ROS bursts activate the Adenosine Monophosphate-Activated Protein Kinase/mTOR pathway to initiate autophagosome formation, but downregulation of lysosomal membrane protein LAMP2 disrupts autophagosome-lysosome fusion.^40^ Remarkably, melatonin exhibits a unique dual-phase regulatory capacity in this model, blocking ROS-triggered hyperactivation of autophagy while promoting autophagosome degradation, ultimately reducing LC3-II and p62 levels. Although existing literature reports that melatonin regulates autophagic flux integrity *via* mTOR-dependent pathways^41^ and reduces p62 accumulation by restoring lysosomal function in heat stress models, the specific molecular mechanisms underlying melatonin-mediated restoration of autophagic flux in this study warrant further investigation.

Ferroptosis is a form of programmed cell death driven by iron-dependent lipid peroxidation, characterized by abnormal iron accumulation and excessive generation of lipid ROS.^42^, ^43^ In porcine Sertoli cells, heat stress induces OS, generating large amounts of lipid peroxides that trigger ferroptosis.^44^ Interestingly, melatonin has been shown to alleviate membrane lipid oxidative damage by reducing iron ion concentration in a pig skin model,^45^ suggesting its potential role in regulating ferroptosis. RNA-seq analysis in this study revealed that ferroptosis plays a critical role in heat stress-induced testicular injury, while melatonin intervention effectively alleviated heat stress-triggered ferroptosis in spermatocytes (Figure 5(a)). The core driver of ferroptosis, Fe²⁺, catalyzes lipid peroxidation through the Fenton reaction, disrupting cellular iron homeostasis. Ferro Orange staining results revealed that melatonin significantly reduced Fe²⁺ accumulation (Figure 5(b)). In a ferroptosis-mediated OS model of chronic heart failure in rats, myocardial tissues exhibited upregulated expression of p53 and COX2 alongside downregulated GPX4.^46^ Similarly, heat stress in this study elevated p53 and COX2 levels, which were attenuated by melatonin treatment, indicating that melatonin suppresses ferroptosis by blocking DNA damage response and inflammatory amplification pathways (Figure 5(e)). In antioxidant regulation, NRF2—a master transcription factor in OS—activates protective genes such as HO-1 through nuclear translocation.^47^ However, this study found that persistent activation of the NRF2/HO-1 axis under heat stress led to iron metabolism imbalance, manifested as accelerated heme degradation by HO-1 and subsequent free iron release, forming a vicious cycle of "antioxidant defense-iron overload." This mechanism parallels the aberrant NRF2/HO-1 activation observed in cadmium-exposed testicular models.^48^ Melatonin downregulated NRF2 and HO-1 expression, reducing iron accumulation while maintaining basal antioxidant capacity (Figure 5(h)). This bidirectional regulatory strategy echoes its antiferroptotic effects *via* the SIRT6/p-NRF2/GPX4 pathway in cataract models.^18^, ^47^ GPX4, a key ferroptosis inhibitor, specifically reduces phospholipid hydroperoxides to preserve membrane integrity.^49^ Here, heat stress decreased GPX4 expression, which was restored by melatonin (Figure 5(h)). Building on prior studies, melatonin likely reduces iron overload by suppressing the NRF2/Slc7a11 axis,^50^, ^51^ directly scavenges lipid ROS to alleviate GPX4 substrate burden, and enhances GPX4 transcriptional activity *via* SIRT6-dependent epigenetic regulation.^52^ This study elucidates that melatonin exerts multitarget intervention against heat stress-induced testicular ferroptosis by coordinately regulating the p53/COX2 inflammatory axis, NRF2/HO-1 iron metabolism axis, and GPX4 antioxidant axis. These findings provide novel theoretical insights into the mechanisms of male infertility and clinical strategies for its mitigation.

## Conclusion

This study demonstrates that melatonin effectively alleviates heat stress-induced oxidative damage in male germ cells through coordinated regulation of apoptosis, autophagy, and ferroptosis pathways. Both in vivo and in vitro experiments confirm that melatonin enhances antioxidant defenses (*via* elevated SOD/CAT activity and reduced MDA levels), suppresses apoptosis (by inhibiting Caspase-3/Bax), and restores autophagic balance (*via* LC3-II/p62 modulation). Crucially, melatonin counteracts ferroptosis—a key driver of testicular injury—by downregulating the NRF2/HO-1 axis, reducing Fe²⁺ accumulation and lipid peroxidation, while restoring GPX4 expression. These mechanisms collectively preserve spermatocyte survival, promote testicular repair, and restore fertility potential postheat stress. Our findings highlight melatonin’s therapeutic potential for heat-related male infertility through multitarget intervention in OS, autophagy dysregulation, and iron metabolism imbalance.

## Materials and methods

### Animals

Eight-week-old male C57BL/6J mice were purchased from the Experimental Animal Center of Ningxia Medical University. Animals were fed ad libitum and had free access to food and water. They were housed under conditions of constant temperature (21 ± 2 °C) and a 12-h light/dark cycle. All mice were acclimated for 3 days before the experiment. The experiment was approved by the Animal Care and Use Committee of Ningxia Medical University.

Testicular heat stress induction and melatonin intervention eight-week-old male C57BL/6J mice were subjected to a single heat stress treatment at 43 °C for 15 min. Prior to heat stress, the testes were confirmed to have fully descended into the scrotum. Specifically, anesthetized mice had their lower bodies (including hindlimbs and scrotum) immersed in a 43 °C water bath for 15 min. After treatment, mice were dried and returned to the animal facility with normal food and water provided. Mice were randomly divided into four groups: control (Con), heat stress 1 day, heat stress 2 days, and heat stress 7 days. Testicular samples were collected at each time point for subsequent analyses.

For melatonin intervention experiments, the experiment involves 20 male C57BL/6J mice (6–8 weeks old), randomly divided into 4 groups (n = 5). The control group and heat stress (43 °C heat) group received an intraperitoneal injection of 10 mL/(kg·d) 0.9% saline for 10 days. The 43 °C heat+MT and MT groups receive an intraperitoneal injection of 20 mg/(kg·d) melatonin for 10 days. On the third day after treatment, the mice in the 43 °C heat and 43 °C heat+MT groups are anesthetized with 4% chloral hydrate and subjected to a single heat stress session in a 43 °C water bath for 15 min.

### Cellular heat stress experiment

The GC-2spd (ts) cells from mice were divided into four groups: the Control group (cultured under standard conditions for 30 h), the MT group (cultured under standard conditions for 24 h, then switched to a complete medium containing 10 µmol/L melatonin for an additional 6 h), the 43 °C heat group (cultured under standard conditions for 36 h, then exposed to heat stress at 43 °C in an incubator for 2 h, followed by a 4-h recovery period), and the 43 °C heat + MT group (treated with 1 µmol/L melatonin followed by heat stress).

### Histological analysis of testes

Testicular tissues were fixed in 4% paraformaldehyde for 24 h, dehydrated in graded ethanol, and embedded in paraffin. Tissue sections were prepared and stained with H&E. Histological changes were observed under a light microscope.

### Measurement of MDA levels and activities of SOD and CAT

Testicular tissues were washed with phosphate-buffered saline solution and weighed. After the weights were recorded, homogenization was immediately performed using a tissue homogenizer on ice and centrifuged. The supernatants were used for the measurements. MDA, SOD, and CAT assays were performed using a spectrophotometer (Jianglai Biotechnology, Shanghai, China). The analysis was performed according to the manufacturer’s instructions.

### Immunohistochemistry

Paraffin-embedded testicular sections underwent deparaffinization and hydration, antigen retrieval, and blocking with 5% normal goat serum. The sections were incubated overnight at 4 °C with the following primary antibodies: anti-Sycp3 (1:200, Proteintech), anti-Bax (1:200, Cell Signaling Technology), and anti-Gpx4 (1:200, Affinity) 4 °C, and then biotinylated rabbit anti-mouse antibody was added. Immunoreactivity was detected by indirect immunoperoxidase staining (Vector Labs, Newark, CA, USA) with DAB. Images were obtained under a microscope.

### Western blot analysis

The testicular tissues of mice in each group were extracted with RIPA lysis buffer, and the protein concentration was determined using the bicinchoninic acid assay. The total protein from each group was added to the sample buffer for PAGE. Total proteins were separated by electrophoresis on 10–12.5% SDS-PAGE gels. After protein separation, samples were transferred to PVDF membranes, blocked, and incubated with primary antibodies. The primary antibodies included anti-P62, anti-LC3-I, anti-LC3-II, anti-P53, anti-COX2, and anti-Caspase-3 (Abcam), Anti-Bax, anti-Bcl-2, anti-HO-1, and anti-GPX4 (Affinity), Anti-β-actin (1:5000, Proteintech) and anti-GAPDH (1:2000, Bioss). The appropriate horseradish peroxidase-conjugated secondary antibodies were diluted 1:20,000 in 1× phosphate-buffered saline and added to the membranes for 1 h at room temperature. The protein bands were visualized using an enhanced chemiluminescent reagent. ImageJ software was used to analyze the band intensities. β-actin was used as an internal control.

### TUNEL assay

Testicular sections were processed through xylene and graded ethanol, followed by DNase-free proteinase K treatment. Sections were incubated with terminal deoxynucleotidyl transferase at 37 °C for 1 h and counterstained with Hoechst 33342. Fluorescence microscopy was used for observation.

### Flow cytometry

Cell apoptosis was assessed using the Annexin V-FITC/PI apoptosis detection kit (Multisciences, China). After 24-h treatment, cells were resuspended in a binding buffer with Annexin V-FITC and PI, incubated in the dark for 5 min, and analyzed using a flow cytometer (EPICS ALTRA, Beckman Coulter, USA). Data were analyzed using (Expo32 v1.2 software, Beckman Coulter, Inc., USA).

### RNA sequencing analysis

RNA purity, concentration, and integrity were assessed to ensure suitability for RNA-Seq. DEGs were identified using DESeq2 software, Bioconductor Project, with thresholds of False Discovery Rate < 0.05 and fold change ≥ 2. Gene ontology and KEGG enrichment analyses were performed for functional annotation.

### Ferro orange staining

Fe²⁺ levels in spermatocytes were detected using Ferro Orange kits (Dojindo, Japan). Cells were seeded in 6-well plates and cultured overnight. After serum-free medium replacement, cells were treated with 100 µmol/L ferrous ammonium sulfate for 30 min, washed, and incubated with Ferro Orange (1 µmol/L) and 2,2′-bipyridyl (100 µmol/L) for 30 min. Cells were observed under a confocal microscope.

### Statistical analysis

All analyses were performed using Prism 8 software (GraphPad, San Diego, CA, USA). When the distribution of data was not normal, a Mann–Whitney U test was used for analysis. And multi-group data were analyzed by one-way Analysis of Variance followed by Bonferroni’s post hoc test. A *P*-value < 0.05 was considered statistically significant.

## Ethics statement

All animal experiments were approved by the Animal Care and Use Committee of Ningxia Medical University and conducted in accordance with institutional guidelines for the ethical use of laboratory animals.

## Funding and support

This work was supported by the Key Research and Development Program of the Ningxia Hui Autonomous Region (2021BEG02029) and the National Natural Science Foundation of China (82260634).

## CRediT authorship contribution statement

**Jia Hua:** Visualization, Validation, Supervision, Project administration. **Ma Wenzhi:** Supervision, Resources, Project administration, Funding acquisition, Formal analysis. **Wang Jia:** Investigation, Formal analysis, Data curation. **Lei Yi Ping:** Writing – review & editing, Writing – original draft, Conceptualization.

## Additional Notes

None of the authors hold patents or intellectual property related to the findings of this study.

Any external affiliations or commitments have not influenced the results presented here.

## Institutional Review Board Statement

The experiments using mice were approved by the ethics committee of Ningxia Medical University, and all animal care and experiments were carried out in accordance with the institutional ethical guidelines for animal experiments.

## Informed Consent Statement

Not applicable.

## Declarations of interest

The authors declare that they have no known competing financial interests or personal relationships that could have appeared to influence the work reported in this paper.

## Data Availability

The authors do not have permission to share data.
