# Corrigendum to “Melatonin ameliorates heat stress-induced oxidative apoptosis in mouse spermatocytes *via* autophagy and ferroptosis pathways” [*Cell Stress Chaperones*. 2025;30:100078]

**DOI:** 10.1016/j.cstres.2025.100087

**Published:** 2025-06-26

**Authors:** Yi-Ping Lei, Jia Wang, Peng-Luo Yin, Hua Jia, Wen-Zhi Ma

**Affiliations:** Key Laboratory of Fertility Preservation and Maintenance of the Ministry of Education, Key Laboratory of Reproduction and Genetics of Ningxia Hui Autonomous Region, School of Basic Medical Science, Ningxia Medical University, Yinchuan 750004, China

The authors regret that the Acknowledgments section was omitted from the published article.

The Acknowledgments section should read:

Yi-Ping Lei and Jia Wang contributed equally to the experiments, data analysis, and manuscript preparation, and should therefore be designated as co-first authors. Peng-Luo Yin was responsible for the experimental design and data analysis. H.J. participated in the design of the study and edited the manuscript. W.M. contributed to the conception, supervision, and editing of the manuscript. All authors have read and agreed to the published version of the manuscript.

The authors would like to apologize for any inconvenience caused.

